# Accurate Detection and Evaluation of the Gene-Editing Frequency in Plants Using Droplet Digital PCR

**DOI:** 10.3389/fpls.2020.610790

**Published:** 2020-12-14

**Authors:** Cheng Peng, Ming Zheng, Lin Ding, Xiaoyun Chen, Xiaofu Wang, Xuping Feng, Junmin Wang, Junfeng Xu

**Affiliations:** ^1^State Key Laboratory Breeding Base for Zhejiang Sustainable Pest and Disease Control, Institute of Quality and Standard for Agro-Products, Zhejiang Academy of Agricultural Sciences, Hangzhou, China; ^2^Key Laboratory of Biology and Genetic Improvement of Oil Crops, Oil Crops Research Institute of the Chinese Academy of Agricultural Sciences, Ministry of Agriculture, Wuhan, China; ^3^College of Biosystems Engineering and Food Science, Zhejiang University, Hangzhou, China; ^4^Institute of Crops and Nuclear Technology Utilization, Zhejiang Academy of Agricultural Sciences, Hangzhou, China

**Keywords:** gene editing, CRISPR/Cas, accurate detection, dPCR, quantity analysis

## Abstract

Gene-editing techniques are becoming powerful tools for modifying target genes in organisms. Although several methods have been reported that detect mutations at targeted loci induced by the CRISPR/Cas system in different organisms, they are semiquantitative and have difficulty in the detection of mutants in processed food samples containing low initial concentrations of DNA and may not accurately quantify editing frequency, especially at very low frequencies in a complex polyploid plant genome. In this study, we developed a duplexed dPCR-based method for the detection and evaluation of gene-editing frequencies in plants. We described the design, performance, accurate quantification, and comparison with other detection systems. The results show that the dPCR-based method is sensitive to different kinds of gene-editing mutations induced by gene-editing. Moreover, the method is applicable to polyploid plants and processed food samples containing low initial concentrations of DNA. Compared with qPCR and NGS-based methods, the dPCR method has a lower limit of detection (LOD) of the editing frequency and a better relationship with the expected editing frequency in detecting the edited region of gene-edited rice samples. Taken together, the duplexed dPCR assay is accurate and precise, and it will be a powerful tool for the detection and evaluation of gene-editing frequencies in plants in gene-editing technology.

## Introduction

CRISPR-Cas systems have been extensively adapted and used for genome editing and other targeted modifications in organisms (Knott and Doudna, 2018). Compared with traditional *Agrobacterium* mediated T-DNA transgenic method that depends on random recombination or integration in plants, location-specific double strand breaks (DSBs) created by Cas nuclease, which are repaired predominantly by error-prone non-homologous end joining (NHEJ) machinery. The outcome of repair is unpredictable, which may lead to a variety of substitutions or insertion, or deletion (indels) mutations, most frequently indel mutants with only 1 bp variation (Pan et al., 2016; Zhu et al., 2017).

At present, several methods have been reported to detect mutations at targeted loci induced by CRISPR/Cas system in different organisms, including polymerase chain reaction (PCR)-based assay (Kim et al., 2014; Peng et al., 2018), T7 Endonuclease I (Li et al., 2013; Vouillot et al., 2015), high resolution melting curve analysis (HRM) (Thomas et al., 2014), and NGS-based methods (You et al., 2018; Liu et al., 2019). Although these methods have proved to be applicable in diploid plants, the methods are semiquantitative and do not easily detect the processed food samples containing low initial concentrations of DNA or accurately quantify editing frequency, especially at a very low frequency in a complex polyploidy plant genome. It is also worth noting that more and more novel gene-editing tools have been developed to make it easier to modify genomes without being restricted by proto-spacer adjacent motif (PAM), such as base editing and prime editing. Although these new technologies bring many advantages, the editing efficiency in plants is still very low at present (Lin et al., 2020; Tang et al., 2020). Thus, it is a great challenge to the existing detection and evaluation methods to accurately quantify a limited number of mutations using CRISPR/Cas as the development of the gene-editing technology in plants.

Droplet digital PCR (dPCR) is a breakthrough technology that relies on partitioning individual amplifications into separate compartments, as well as the detection of their endpoint amplification products. It provides ultrasensitive and absolute nucleic acid quantification without a standard curve (Hindson et al., 2011; Suo et al., 2020). Up to now, dPCR detection in plants has achieved wide usage in the area of detection of the presence of transgenes within food samples (Dobnik et al., 2015; Zhu et al., 2016; Bogozalec Kosir et al., 2019). Collectively, these data show that dPCR is an ultrasensitive method and has a lower error rate than the other PCR-based method. It suggests that dPCR may permit accurate detection of gene editing events.

In this study, it was first reported on duplexed dPCR for the detection of gene-editing plants and related processed food products. We detail the design, performance, quantity analysis, and comparison with other detection systems. Taken together, the duplexed dPCR assay is an accurate, precise, and adaptable in the detection of multiple copies of a gene in a complex genome, even processed food containing low levels of DNA.

## Materials and Methods

### Plant Materials

We previously created gene-edited rice, and gene-edited rapeseed plants were provided by Dr. Zheng Ming (Supplementary Table 1). The target sequences were designed using the web tool CRISPR-P (Lei et al., 2014).

### DNA Extraction

Plant genomic DNA was prepared using a QIAGEN DNeasy Plant Mini Kit (QIAGEN, Germany) according to the manufacturer’s protocol. The DNA was quantified using a NanoDrop 1,000 instrument (Thermo Scientific, United States). The integrity of the extracted DNA was further characterized by agarose gel electrophoresis. All of the DNA templates were diluted to 10 ng/μl and then stored at −20°C until use.

### Sample Preparation

For analysis of low initial concentrations of DNA, the heterozygous gene-editing DNA samples were diluted with water, and the serial DNA samples (10, 5, 0.4, 0.08, and 0.016 ng/μl) were measured using Qubit^®^2.0 (Life Technologies, United States) and prepared. For analysis of the low editing frequency, the serially homozygous mutant DNA samples were mixed with WT (mutant DNA ranging were 50, 25, 10, 5, 1, 0.5, and 0.1%).

### Primers and Probes Design

Reference genes and probes (Table 1) were chosen based on those previously published for use in dPCR (Collier et al., 2017). Primers and probes at the mutation positions are designed using Primer Express Software 3.0, following the manufacturer’s instructions. In addition, the following principles were observed: the primers must span the mutant positions, and the probes should be in PAM region. In order to maintain the sensitivity of the probes for the mutations, it is better to mark the region for the 5′ end of the probes. The candidate pairs of primers were also tested with conventional endpoint PCR to confirm that a single PCR product of the correct size was produced. The reference gene probes were 5′ HEX-labeled, and the mutation-site-specific probes were 5’ FAM-labeled. Both types of probes were quenched with BHQ or MGB at the 3′ end (Sangon BioTech, China). The primers and probes used in this study are listed in Table 1.

**TABLE 1:** The primers and probes used in dPCR.

Species	Gene names	Primer and probe name	Sequence
*Oryza sativa*	*Os06g0623700*	F-primer	TCGCGCTCATTGTCTTCCT
		R-primer	TGGTCTTGAACATTCTCGTTGTG
		Probe-FAM	CTACTGCCGCCGCC
*Oryza sativa*	LOC_Os2g42314*	F-primer	CCTTCGGAGACACCTTTTGA
		R-primer	TTGAAATGCACATTCGGGTG
		Probe-HEX	CTCCTTCCTCCGCAAGTTCGC
*Brassica napus*	*BnaA03g22900D/BnaC03g26960D*	F-primer	CGACCTTCCTGGTCCGTACTC
		R-primer	GCTTGGCAAGAACGGAGAAG
		Probe-FAM	TTGCCCATGCTGGCT^*a*^
*Brassica napus*	BnaA06g36310D/BnaC07g48660D*	F-primer	GGCCAGGGCTTCCGTGAT
		R-primer	CCGTCGTTGTAGAACCATTGG
		Probe-HEX	AGTCCTTATGTGCTCCACTTTCTGGTGCA

^*a*^Using a MGB probe; *indicating endogenous reference genes.

### dPCR

Droplet digital PCR assays consisted of the following components (final concentrations in 20 μl total reaction volume): 10 μl ddPCR SuperMix for Probes (no dUTP) (Bio-Rad, United States), 450 nM of each primer pair (for the endogenous reference gene and the mutation-site-specific gene) and 250 nM of each probe. The final volume was adjusted with water to 19 μl. Then, 1 μl of template DNA was added. A total of 20 μl of this mixture was placed into a cell of a BioRad DG8^TM^ cartridge, and 70 μl of droplet generator oil was added to this well. The cartridge was placed into a QX200 droplet generator (Bio-Rad, United States) to generate the droplets. The droplets were transferred to a 96-well PCR plate. After heat-sealing with a foil seal, the PCR plate was placed in a 7,500 Real-time PCR system and amplified with the following cycling conditions: 95°C for 10 min, 40 cycles of 94°C for 10 s and the designated temperature (58 or 68°C) for 60 s for annealing and extension, 10 min at 98°C for reaction termination, and cooled to 4°C. Following amplification, the plate was placed into the QX200 droplet reader (Bio-Rad, United States) for data analysis. The wild type and homozygous mutant were definitively able to be distinguished by a 2-dimensional view of the dPCR analysis. For heterozygous mutants, the concentration of the mutant droplets and the wild-type droplets were analyzed and generated using Bio-Rad QuantaSoft^TM^ software (v1.7.4) with default settings for threshold determination to distinguish positive and negative droplets. The mutation frequency of gene editing was quantified by the ratio of mutant droplets (only HEX positive droplets) to wild-type droplets (HEX/FAM double positive droplets). In each experiment, at least three biological replicates were carried out.

### Quantitative Real-Time PCR

Quantitative real-time PCR was performed on a 7,500 Real-Time PCR Cycler (Life Technologies AB, United States) using the FastStart Universal Probe Master (Roche, Switzerland) with ROX reference dye according to the manufacturer’s instructions. The qPCR assays were analyzed according to our previous study (Peng et al., 2018). In each experiment, at least three biological replicates were carried out.

### NGS-Based Sequencing

The PCR primers (Table 2) were designed to amplify a product flanking the target mutation sites using a Nested-PCR strategy, and unique sample-specific barcodes were attached to the PCR products. High-throughput sequencing was performed using an Illumina HiSeq platform (Illumina, United States) by the Novogene Bioinformatics Institute, Beijing, China. The concentration of the libraries was initially measured using Qubit^®^2.0 (Life Technologies, United States). Gene-editing frequency was analyzed using the Hi-TOM program for high-throughput mutation sequence decoding^1^.

**TABLE 2:** The primers used in NGS-based sequencing.

Primer name	Sequence	Describe
Os-HI-F	ggagtgagtacggtgtgcAGCGTACGTGCAGTGCAGCCAA	for rice
Os-HI-R	gagttggatgctggatggAGTGATCCGCCGAGGTCCAGAT	
Bn-HI-F	ggagtgagtacggtgtgcTACTCAATGTCCCCGGTCCAAC	for rapeseeds
Bn-HI-R	gagttggatgctggatggAACTGAACCAAAATCCAAATATCCC	

## Results

### Design a dPCR Platform for Detection of Gene-Editing Rice

**FIGURE 1:**
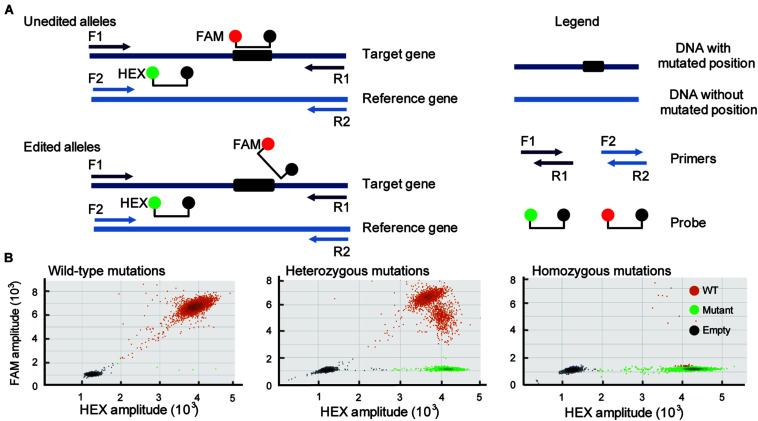
Development of a dPCR platform for detection of gene editing. **(A)** Schematic illustrates the dPCR-based assay (primers F1 and R1 span the DNA with mutated position; primers F2 and R2 span the DNA without mutated position). Amplicons are detected by the activation of amplicon-specific fluorescent probes. A FAM-labeled probe locates at the mutation position, and a HEX-labeled probe locates without mutation position. **(B)** Three typical droplet plots of the wild-type mutations (WT; right panel), heterozygous mutations (middle panel), and homozygous mutations (right panel).

CRISPR/Cas9-induced mutations are predictable, and most of the mutations occur within 3 bases upstream of the 5′ end of the PAM (van der Oost, 2013). Based on this knowledge, duplexed dPCR was developed. In this dPCR system, there are two pairs of primers and probes. One pair of primers spans the mutation position, and another pair does not (Figure 1A). Amplification is detected using primer-pair specific probes (6-carboxyfluorescein, FAM-labeled probe, mutation-site-specific probe; 5-hexachloro-fluorescein, HEX-labeled probe, reference gene probe). Thus, a CRISPR/Cas9-induced mutation will disrupt the binding of the FAM probe but will not affect that of the HEX (Figure 1A). The FAM-labeled probe was used to assess the amount of mutant alleles, and the HEX-labeled probe was used to assess the total amount of alleles present in the samples. As a result, in a two-dimensional view of the dPCR analysis, droplets containing both fluorescent signals are wild-type amplicons, while droplets containing HEX-positive but FAM-negative signals are mutant amplicons. The amplification of heterozygous mutation contained either mutant droplets or wild-type droplets (Figure 1B). We can also use the ratio of mutant droplets (only HEX-positive droplets) to wild-type droplets (HEX/FAM-double positive droplets) to quantify the mutation frequency of gene editing. To establish the dPCR-based method to detect the mutations in gene-editing plants, we first evaluated the performance of the method using the three typical gene-editing rice samples. They are respectively, wild-type, homozygous and heterozygous mutations induced by CRISPR/Cas9 in the rice *TGW6* gene that we have previously identified by sequencing (Ishimaru et al., 2013; Peng et al., 2018). As expected, the three typical gene-editing rice samples were definitively able to distinguish by 2-dimensional view of the dPCR analysis (Figure 1B).

### The dPCR Platform Can Efficiently Identify Different Kinds of Mutations Induced by Gene Editing

**FIGURE 2:**
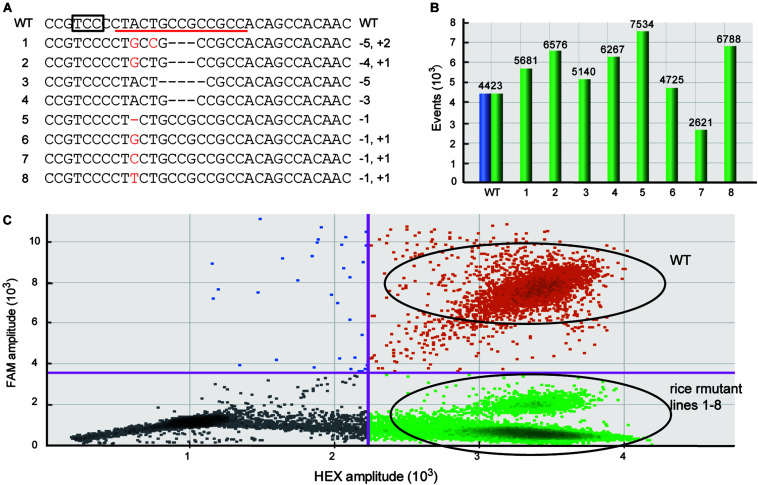
Efficiently detecting different kinds of mutations used dPCR. **(A)** The target sequences of different mutant rice lines. The box and red line indicate the PAM and FAM-labeled probe, respectively. **(B)** The total number of events displayed using software to correct for Poisson distribution. Blue bars represent FAM-positive events, and green bars show HEX-positive events. **(C)** The combined droplet plots of different kinds of mutant gene-edited rice lines.

Because the mutations produced by Cas are frequently indel mutants with only 1 bp variation (Pan et al., 2016; Zhu et al., 2017), it is important that the newly developed method can efficiently detect these tiny genetic mutations. To test whether the dPCR method can be sensitive to different kinds of mutation, such as single nucleotide indels, we used the dPCR platform to detect the rice samples containing various types of homozygous mutations verified by sequencing (Figure 2A). These mutations contain not only single nucleotide indels, but also single nucleotide mutations (Figure 2A). The dPCR result shows that in all these samples droplets contained HEX-positive but FAM-negative signals, and there are almost no FAM-positive droplets/while there are thousands of the HEX positive droplets (Figures 2B,C). Thus, the dPCR-based detection method can efficiently identify different kinds of mutations induced by gene editing.

### Ultra-Sensitive Detection of Gene Edited Samples

**FIGURE 3:**
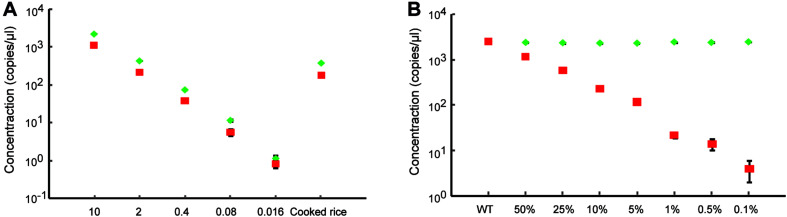
Ultra-sensitive detection of gene-edited samples. **(A)** Detection of heterozygous gene-edited samples containing different initial concentrations of DNA and cooked rice by dPCR. The red square and the green diamond show the concentrations of mutant droplets and wild-type droplets, respectively. All data are show as the mean ± standard deviation (SD) of three replicates. **(B)** Accurate quantification of the gene-editing induced mutation ratio in mixed samples. The red square and the green diamond show the concentrations of mutant droplets and wild-type droplets, respectively. All data are show as the mean ± standard deviation (SD) of three replicates.

**FIGURE 4:**
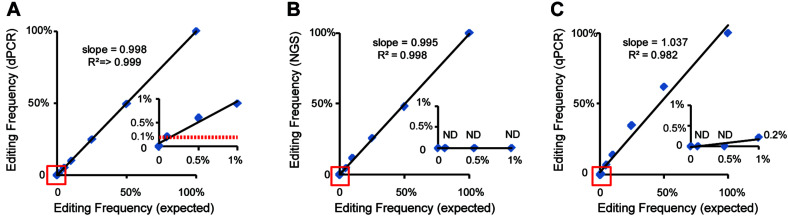
Comparison and analysis of dPCR-, qPCR-, and NGS-based methods. Standard curves show the relationship between expected editing frequency and editing frequency, respectively, measured by **(A)** the dPCR assay, **(B)** the NGS-based method, and **(C)** the qPCR assay. The red lines in panel **(A)** show a good relationship between 0.1% expected editing frequency and which measured by the dPCR assay. ND indicated undetected.

Next, we evaluated the performance of dPCR using the samples containing low initial concentrations of DNA and even processed food samples. A series of the heterozygous DNA samples diluted with water were prepared. The results show that the dPCR method could accurately detect samples with as low as 0.08 ng/μl, but 0.016 ng/μl DNA samples were hard to identify from background noise due to the loss of proper mutation frequency of heterozygous DNA (Figure 3A and Supplementary Figure 1). For processed food samples, the dPCR method also shows good performance to detect our prepared cooked rice samples (Figure 3A and Supplementary Figure 1) indicated that dPCR has a wide range of applications. In order to determine the limitation of detection (LOD) of mutant frequency of dPCR-based assay, we mixed the mutant template (homozygous mutant DNA) and wild-type template in various ratios (with mutant DNA ranging from 50 to 0.1%). The concentration of mutant droplets in mixed samples was gradually reduced along with the reducing amounts of mutant templates while the concentration of wild-type droplets stays roughly the same (Figure 3B and Supplementary Figure 2). The result shows that the dPCR-based method could detect a mutant template at frequencies of 0.1%, with a standard deviation (SD) of 0.021% (Figure 4A). Meanwhile, our results show that dPCR is precise and technical replicates are essentially indistinguishable from one another (Figure 3B). Therefore, dPCR is an ultrasensitive method for detection and evaluation of the gene editing, and it can be applied to detect samples containing low initial concentrations of DNA, such as processed food samples.

### A Comparison of dPCR-, qPCR-, and NGS-Based Methods

At present, qPCR- and NGS-based methods have been shown to be useful for quantitative detection of the mutant frequency induced by gene-editing in plants (Peng et al., 2018; Liu et al., 2019). We further detected and analyzed the same mixed samples using qPCR- and NGS-based methods. Compared with the dPCR method, the results of the qPCR- and NGS-based methods show no positive signals at a rate of mutant templates below 5% (Figures 4A–C and Table 3), suggesting the LOD of qPCR and NGS-based methods were about 5% (Figures 4B,C). The relationship between observed by dPCR assay and expected editing frequency is linear (Pearson’s *R*^2^ > 0.999), while qPCR- and NGS-based methods gave *R*^2^ = 0.982 and 0.998, respectively. Moreover, by using NGS-based methods, we found there is an unexpected mismatch (T- > C) in editing the target region of some mixed samples (Table 3), which might be caused by NGS sample preparation. Taken together, dPCR presents a more accurate and more quantitative way to detect and evaluate gene-editing frequencies compared with the other methods in this CRISPR-edited region.

**TABLE 3:** Detection of gene-editing frequency via the NGS-based method.

Editing frequency (expected)	Sort	Reads number	Ratio	Left variation type	Right variation type	Left variation	Right variation	Editing frequency (NGS)
50%	1	742	51.89%	WT	5D	–	GCCGC	51.89%
	2	688	48.11%	WT	WT	–	–	
25%	1	1144	73.19%	WT	WT	–	–	25.78%
	2	403	25.78%	WT	5D	–	GCCGC	
	3	16	1.02%	WT	SNP	–	T- > C	
10%	1	1303	88.22%	WT	WT	–	–	11.78%
	2	174	11.78%	WT	5D	–	GCCGC	
5%	1	1557	94.54%	WT	WT	–	–	5.46%
	2	90	5.46%	WT	5D	–	GCCGC	
1%	1	1450	100.00%	WT	WT	–	–	0%
0.50%	1	1650	99.04%	WT	WT	–	–	0%
	2	16	0.96%	WT	SNP	–	T- > C	
0%	1	1596	100.00%	WT	WT	–	–	0%

### Using dPCR Platform Detection of Gene-Editing Mutations in Allotetraploid Rapeseed

**FIGURE 5:**
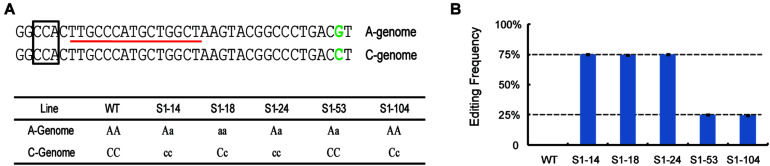
Detection of the editing frequencies in allotetraploid rapeseed using droplet digital PCR. **(A)** The target sequences and genotypes at the target site of gene-edited rapeseeds. The box and red line indicate the PAM- and FAM-labeled probe, respectively. The green colors indicate the SNP differences between the Chr. A03 and C03 genome. **(B)** Detection of editing frequency of different rapeseed lines by dPCR.

To further confirm whether the dPCR method can be used for accurate detection of gene-editing frequency in polyploid organisms, allotetraploid rapeseed (*Brassica napus* L., AACC, *n* = 38) samples were chosen for analysis. We used previously- created 5 gene-edited rapeseed lines that have target gene-editing on different chromosomes; one target was heterozygous on Chromosome A03 or C03, while the other was a homozygous or wild-type mutation by Sanger Sequencing (Figure 5A and Supplementary Table 1; Zheng et al., 2020). Thus the editing frequency of these samples should theoretically be 75% (one genome having homozygous mutation, another heterozygous) or 25% (one genome homozygous wild-type, another heterozygous). As expected, use of the dPCR assay gave editing frequencies of S1–14, S1–18, and S1–24 as 75%, whereas S1–53 and S1–104 were shown to be 25%, which is consistent with the previous result (Figure 5B), suggesting the robustness of our dPCR method in the detection of mutations in polyploid organisms.

## Discussion

CRISPR technology has been widely used in plant gene editing and has great potential for precision breeding (Rodriguez-Leal et al., 2017; Knott and Doudna, 2018). With the development of CRISPR technology, accurate and quantitative detection method for gene-editing plants needs to be developed. On the one hand, new CRISPR technology, such as prime editing that can directly write new DNA information into a specified DNA site, greatly expanding the scope and capabilities of gene editing. However, the editing efficiency of prime editing in plants is still very low at present (Lin et al., 2020), traditional semiquantitative methods are difficult to identify and evaluate. On the other hand, although different countries have different regulatory frameworks for gene-editing crops, edited crops and the final food products may go to the market. Food products are more difficult to detect than plant tissues due to low quality of DNA. Detection methods will need to address these challenges. The T7EI method is widely used in research. That method relies on T7EI and can recognize and digest mismatched heteroduplex DNA; however, these detection methods are known to be semiquantitative (Li et al., 2013; Vouillot et al., 2015). Next generation sequencing is a comprehensive method that can provide more accurate information than dPCR/Sanger sequencing, especially in multiple sgRNA or large indels. However, it presents hurdles in terms of workflow and cost, and NGS sample preparation can introduce mismatches in the target editing region that impact analysis (Table 3; You et al., 2018; Liu et al., 2019). The qPCR method is effective in detecting diploid plants, but its precision is limited, and it depends on the standard curve (Peng et al., 2018). In our research, we have demonstrated that the dPCR-based method could provide absolute quantitative analysis of gene-editing frequencies in plants without a standard curve. Taking advantage of the dPCR platform, our methods are more accurate; moreover, they had a lower LOD compared with qPCR- and NGS-based methods for the detection of the edited region of gene-edited rice samples and might be more suitable for detecting and evaluating the gene-editing frequencies in plants (Figure 4). Furthermore, the dPCR-based method is particularly useful for the detection of gene-edited food (Figure 3A). We have shown that only a small amount of gDNA (as little as 0.08 ng sample loading) is required for detection, which cannot be achieved by other methods. Although dPCR does not provide the mutation information induced by gene editing that could be obtained via future Sanger sequencing, the speed, accuracy, and efficiency of the dPCR method will facilitate the identification of gene-edited plants and their derivatives, in addition to aiding in more accurate analysis to assess the gene-editing frequency.

Some studies have found that the DNA sample used for dPCR analysis should use restriction endonucleases to facilitate the complete and random partitioning of the genomic DNA into droplets (Collier et al., 2017). It is worth noting that in the use of restriction endonucleases to treat genomic DNA, the enzyme should be carefully chosen and validated by testing it with dPCR first. The enzyme chosen should not have recognition sites within either the reference or gene-editing target amplicon sequences. In this study, to make the dPCR method easy to use, we did not use restriction endonucleases to treat genomic DNA. Although several studies suggest that fractionation was necessary in some dPCR applications, it is optional in the use of the dPCR method to detect and evaluate gene-editing in samples.

Nonetheless, dPCR-based methods have common limitations, which may inevitably be affected if the gene-editing target amplicon region contains large deletions or if mutations occur outside of the predicted target area. To overcome this situation, running a regular PCR analysis for an initial screen can be a solution prior to dPCR analysis. However, such situations are rare and not necessarily more advantageous than small, out-of-frame deletions in the predicted region. In addition, compared with previous detection and screening of gene-editing plants using qPCR (Peng et al., 2018), some improvements have been made in this study. The qPCR methods used two differently labeled probes for the detection of the same PCR product. Sometimes it will be difficult to design two probes for one PCR-amplified fragment. The dPCR method presented here employed a validated probe from endogenous reference genes to form a double amplification that made it easier to develop and reduced costs (the endogenous reference probes and primes can also be useful in the detection of another editing target). Here, we used a duplexed dPCR assay to detect gene-editing frequencies in plants as well as processed food samples. Moreover, some studies have shown that the dPCR approach could analyze gene-editing frequencies in animal cells (Mock et al., 2016; Rose et al., 2017), indicating that the dPCR method may have wide applicability. In our study, limited by types of gene-edited plants, two species—rice, a diploid, and rapeseed, a tetraploid—were tested. In the future, more plant species and multiple edited regions or genes need to be tested by dPCR and compared with other methods, which will extend its usage for the research community. In conclusion, the dPCR-based method is accurate and precise, and it will be a powerful, usable tool to detect and evaluate gene-edited plants and their derivatives.

## Data Availability Statement

The original contributions presented in the study are included in the article/Supplementary Material, further inquiries can be directed to the corresponding author/s.

## Author Contributions

CP and JX conceived the project and wrote the manuscript. CP, LD, XC, and XW conducted the experiments. CP, MZ, XF, and JW analyzed the data. All authors contributed to the article and approved the submitted version.

## Conflict of Interest

The authors declare that the research was conducted in the absence of any commercial or financial relationships that could be construed as a potential conflict of interest.

## Footnotes

1 http://www.hi-tom.net/hi-tom/

## Supplementary Material

Supplementary Figure 1 The droplet plots of the samples containing different initial concentrations of DNA.

Supplementary Figure 2 The droplet plots of DNA samples with the different gene-editing frequencies.

Supplementary Table 1 The source of gene-edited plants used in this study.
