# Supplementary material for: Accurate Detection and Evaluation of the Gene-Editing Frequency in Plants Using Droplet Digital PCR
Source: Front Plant Sci. 2020 Dec 14;11:610790. doi: 10.3389/fpls.2020.610790 (PMC7767858; doi:10.3389/fpls.2020.610790)
Supplement: Supplementary Figure 1 — The droplet plots of the samples containing different initial concentrations of DNA. [file Data_Sheet_1.docx]

**Supplementary Figures**

**Figure S1. The droplet plots of the samples containing different initial concentrations of DNA.**

**Figure S2. The droplet plots of DNA samples with the different gene-editing frequency.**

| **Supplementary Table**  **Table S1. The source of gene-edited plants used in this study** | | | |
| --- | --- | --- | --- |
| **Rapeseed lines** | **Genotypes at CRISPR target site** | | **Reference** |
|  | ***BnaA03g22900D*** | ***BnaC03g26960D*** |  |
| WT | WT | WT | Zheng et al., 2020 |
| S1-14 | Heter | Homo |  |
| S1-18 | Homo | Heter |  |
| S1-24 | Heter | Homo |  |
| S1-53 | Heter | WT |  |
| S1-104 | WT | Heter |  |
| **Rice lines** | ***Os06g0623700*** | | Peng et al., 2018 |
| 1 | Homo | |  |
| 2 | Homo | |  |
| 3 | Homo | |  |
| 4 | Homo | |  |
| 5 | Homo | |  |
| 6 | Homo | |  |
| 7 | Homo | |  |
| 8 | Homo | |  |
| All gene-edited plants used in this study have been validated by published literatures.  Notes: The Heter, heterozygous; Homo, homozygous; | | | |
